# Supplementary material for: Obesity-induced galectin-9 is a therapeutic target in B-cell acute lymphoblastic leukemia
Source: Nat Commun. 2022 Mar 3;13:1157. doi: 10.1038/s41467-022-28839-y (PMC8894417; doi:10.1038/s41467-022-28839-y)
Supplement: Supplementary file 1 — Supplementary Information [file 41467_2022_28839_MOESM1_ESM.pdf]

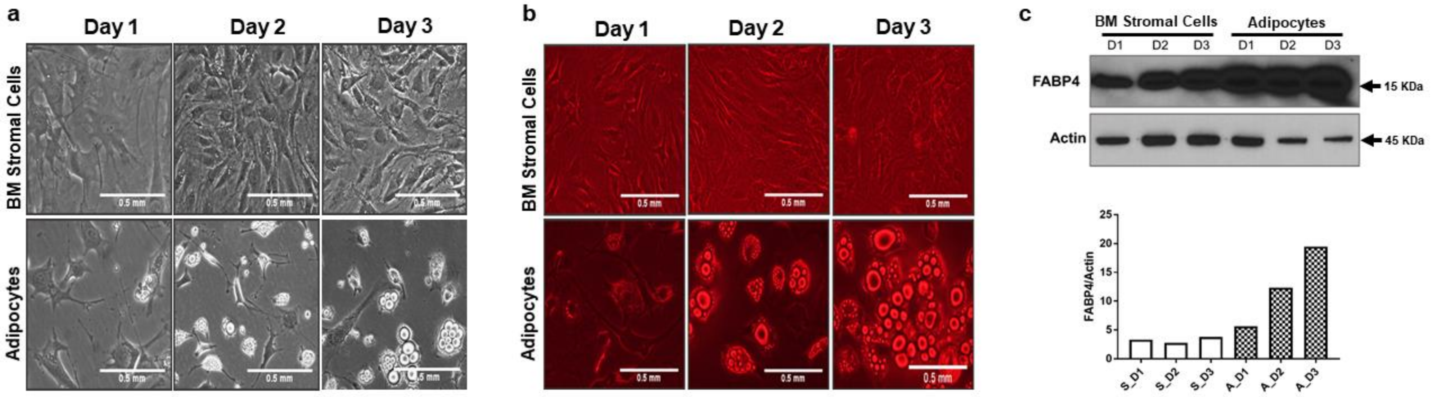

Supplementary Fig. 1. *In vitro*-derived adipocytes accumulate lipid droplets and express high amounts of fatty acid binding protein 4 (FABP4). Bone marrow (BM) stromal cells were cultured in RPMI medium or adipocyte differentiation medium for 1, 2, or 3 days. a, Brightfield microscopy images were obtained. Representative images are shown (n=10 independent experiments). b, The accumulation of lipid droplets during the differentiation process was determined on days 1 through 3 of culture via Nile Red staining and confocal microscopy. c, Fatty acid binding protein 4 (FABP4), which is highly expressed in adipocytes, was detected by immunoblot. Actin is shown as a loading control. Western blot source data are provided in the Source Data file.

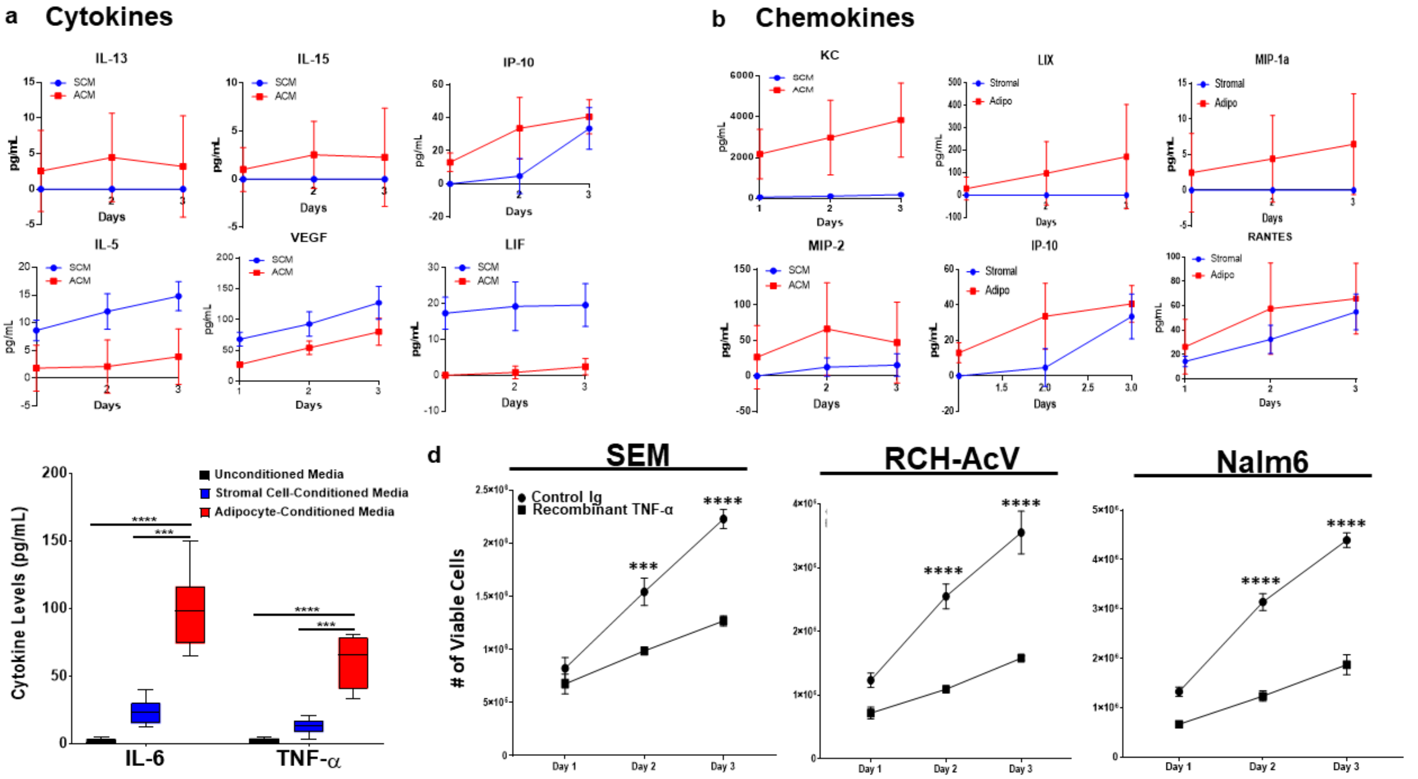

Supplementary Fig. 2. Differentiated adipocytes secreted pro-inflammatory cytokines and chemokines, of which, TNF-α suppresses the proliferation of human B-ALL cells. a,b, Bone marrow stromal cells were cultured in RPMI medium or adipocyte differentiation medium for 1, 2, or 3 days. Supernatants were collected and cytokine and chemokine levels were determined by Luminex analysis. c, IL-6 and TNF-α concentrations in unconditioned medium, stromal cell-conditioned medium, and adipocyte-conditioned medium were determined via ELISA analysis. Box and Whisker plots display the median, 25th, and 75th percentile. The whiskers extend from the minima to the maxima, respectively. d, Human B-ALL cell lines (SEM, RCH-AcV, and Nalm6) were cultured for 3 days in RPMI medium supplemented with control immunoglobulin (control Ig) or recombinant TNF-α. The total number of viable cells was enumerated on days 1 through 3 of culture using trypan blue exclusion assays. Means ± s.d. are shown. ***p<0.001 and ****p<0.0001, n=4 for c-d, one-way ANOVA with Tukey's post-test was used to determine significance in c and two-sided Student's t-test was performed relative to the lean control for each time point in d.

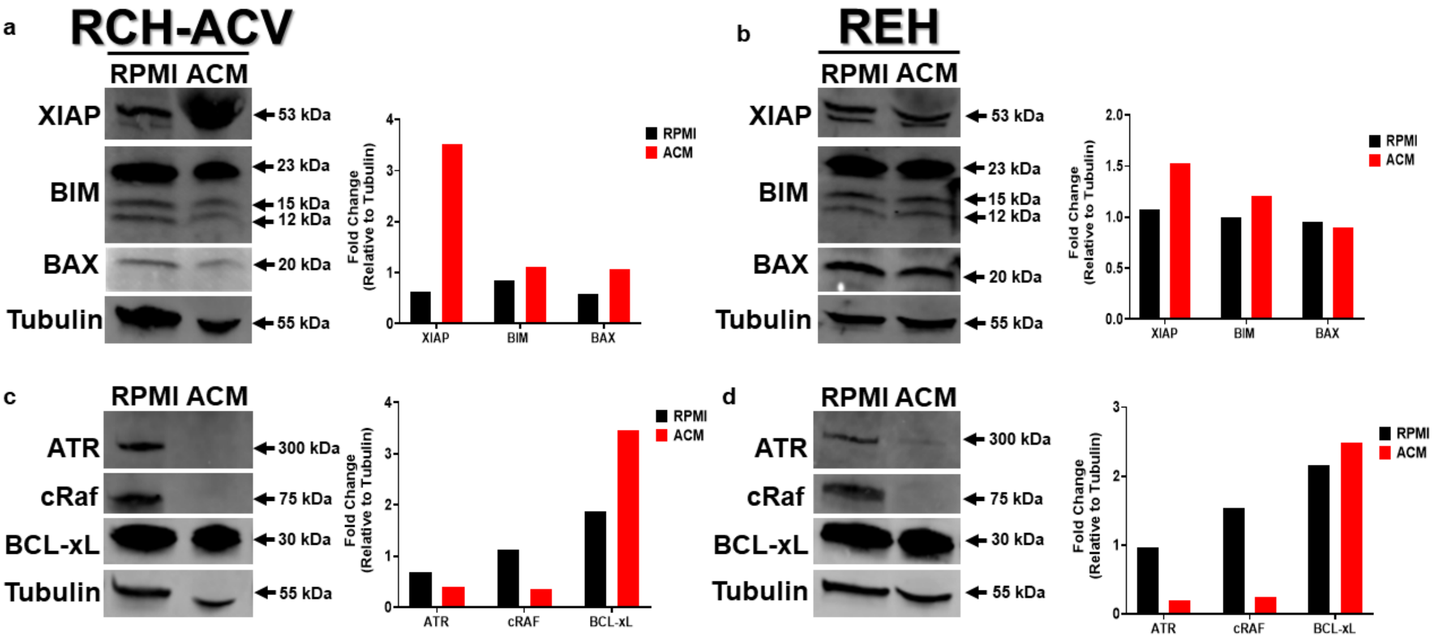

Supplementary Fig. 3. Adipocyte-secreted factors upregulate pro-survival proteins and downregulated DNA damage sensing proteins in human B-ALL cells. Human B-ALL cell lines (RCH-AcV in a,c and REH in b,d) were cultured in unconditioned medium (RPMI) or adipocyte-conditioned medium (ACM) for 24 hours and levels of proteins involved in cellular survival (X-linked inhibitor of apoptosis [XIAP], BIM, BAX, BCL-xL) and DNA damage sensing (ATR and cRAF) were quantified using western blot analysis. Signal intensity was determined using the ImageJ software and graphed as fold changes relative to the internal tubulin control. A representative experiment is shown from n=3 independent experiments. The black arrows in the BIM blots (BIM_L and BIM_S splice variants) indicate the more potent isoforms associated with cytotoxicity. The proteins chosen for analysis represent changes between these groups identified in our RNA-sequencing results. Western blot source data are provided in the Source Data file.

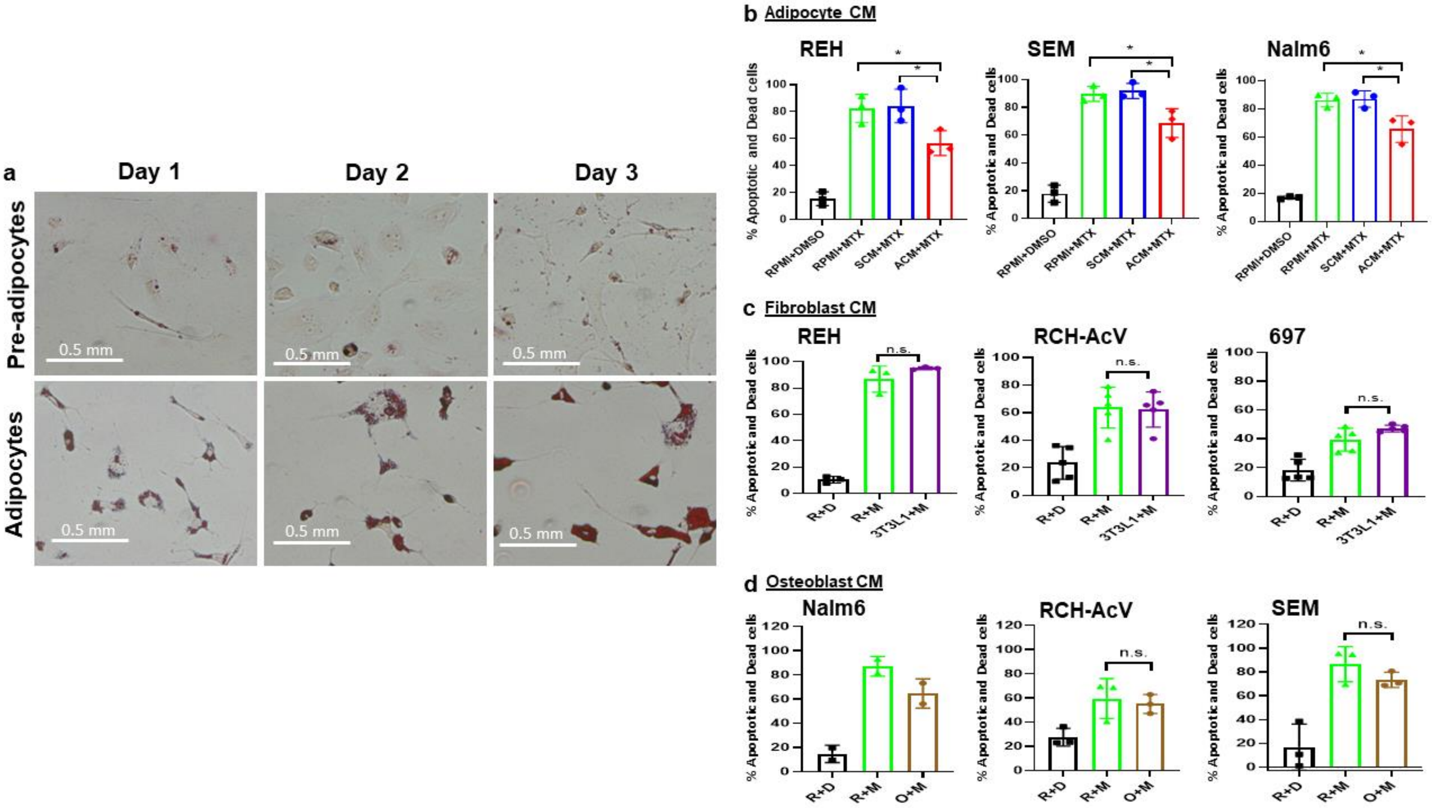

Supplementary Fig. 4. Primary adipocyte conditioned medium (CM), but not fibroblast- or osteoblast-conditioned medium (CM), confers chemoresistance to human B-ALL cells. a, Peripheral fat was harvested from the posterior subcutaneous fat depot and digested with collagenase II to enrich for preadipocytes. Preadipocytes were then cultured in 15% DMEM/F12 medium until cells reached 50-60% confluency. Once this density of preadipocytes was reached, cells were cultured in insulin oleate medium to induce full adipocyte maturation as previously described. Primary images are shown. b-d, Human B-ALL cell lines (REH, SEM, and Nalm6) were preconditioned for 24 hours with unconditioned medium (10% RPMI), adipocyte-conditioned medium (b), fibroblast-conditioned medium (c), or osteoblast-conditioned medium (d) prior to treatment with vehicle (DMSO) or methotrexate (MTX) for an additional 2 days (cells remained in CM during chemotherapy treatment). The percentage of apoptotic and dead cells on day 3 of culture was determined using Annexin-V/ PI staining followed by flow cytometric analysis. Means ± s.d. are shown (**p*<0.05, one-way ANOVA with Tukey's post-test). For b-d, n=3 independent experiments (with dots representing the average of technical replicates for each experiment).

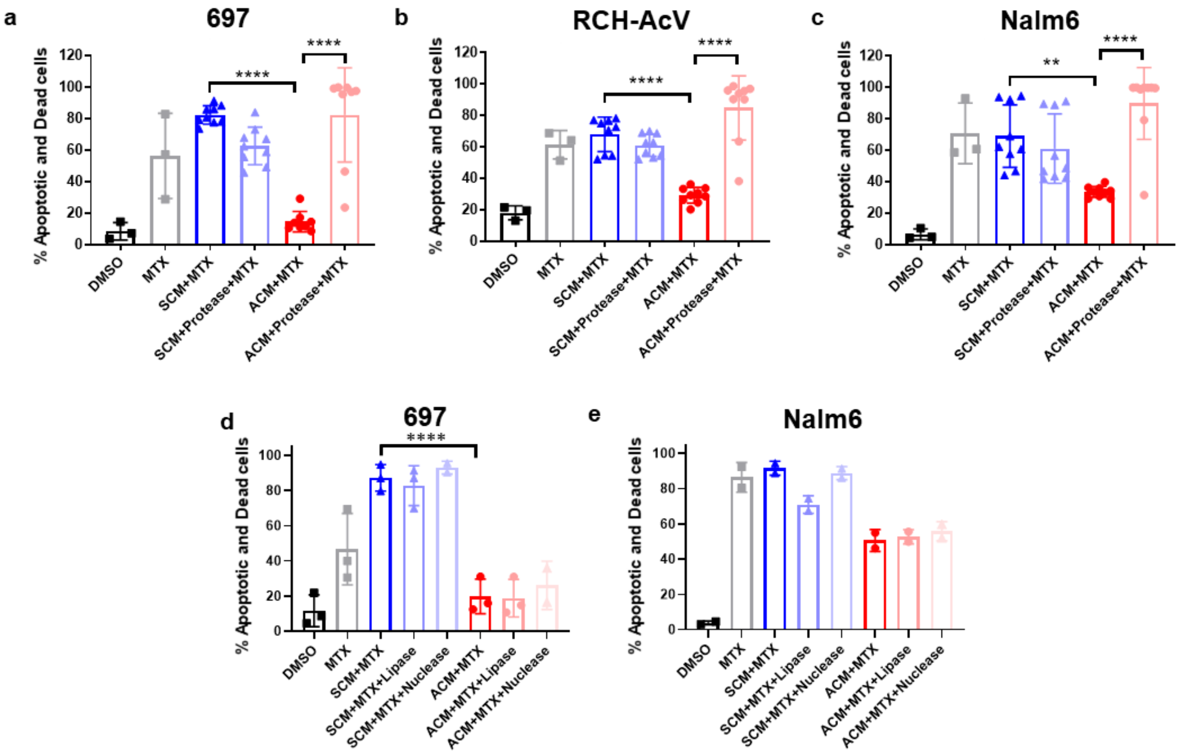

Supplementary Fig. 5. Chemoresistance in human B-ALL is mediated by adipocyte-secreted proteins. a-e, Human B-ALL cell lines (697, RCH-AcV, and Nalm6) were preconditioned for 24 hours with lipase, nuclease, or protease-treated unconditioned medium (10% RPMI), SCM, or ACM prior to treatment with methotrexate (MTX) or vehicle (DMSO) for an additional 2 days (cells remained in CM during MTX treatment). The percentage of apoptotic and dead cells on day 3 of culture was determined using Annexin-V/ PI staining followed by flow cytometric analysis. Means ± s.d. are shown (***p*<0.01 and *****p*<0.0001, n=3 independent experiments, one-way ANOVA with Tukey's post-test).

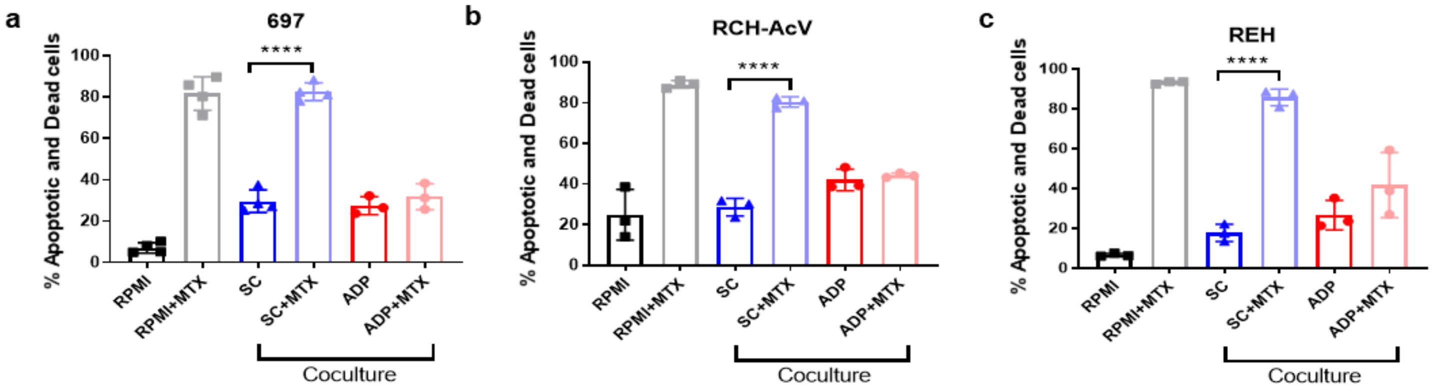

Supplementary Fig. 6. Chemoresistance in human B-ALL is mediated through direct contact with adipocytes. a-c, Human B-ALL cell lines (697, RCH-AcV, and REH) were cultured alone (RPMI), with BM stromal cells (SC), or with adipocytes (ADP) for 24 hours prior to treatment with MTX for an additional 2 days. Apoptotic and dead cells were detected on day 3 of culture by flow cytometric analysis. Means $\pm$ s.d. are shown (**$p<0.01$ and ****$p<0.0001$, n=3 independent experiments, one-way ANOVA with Tukey's post-test).

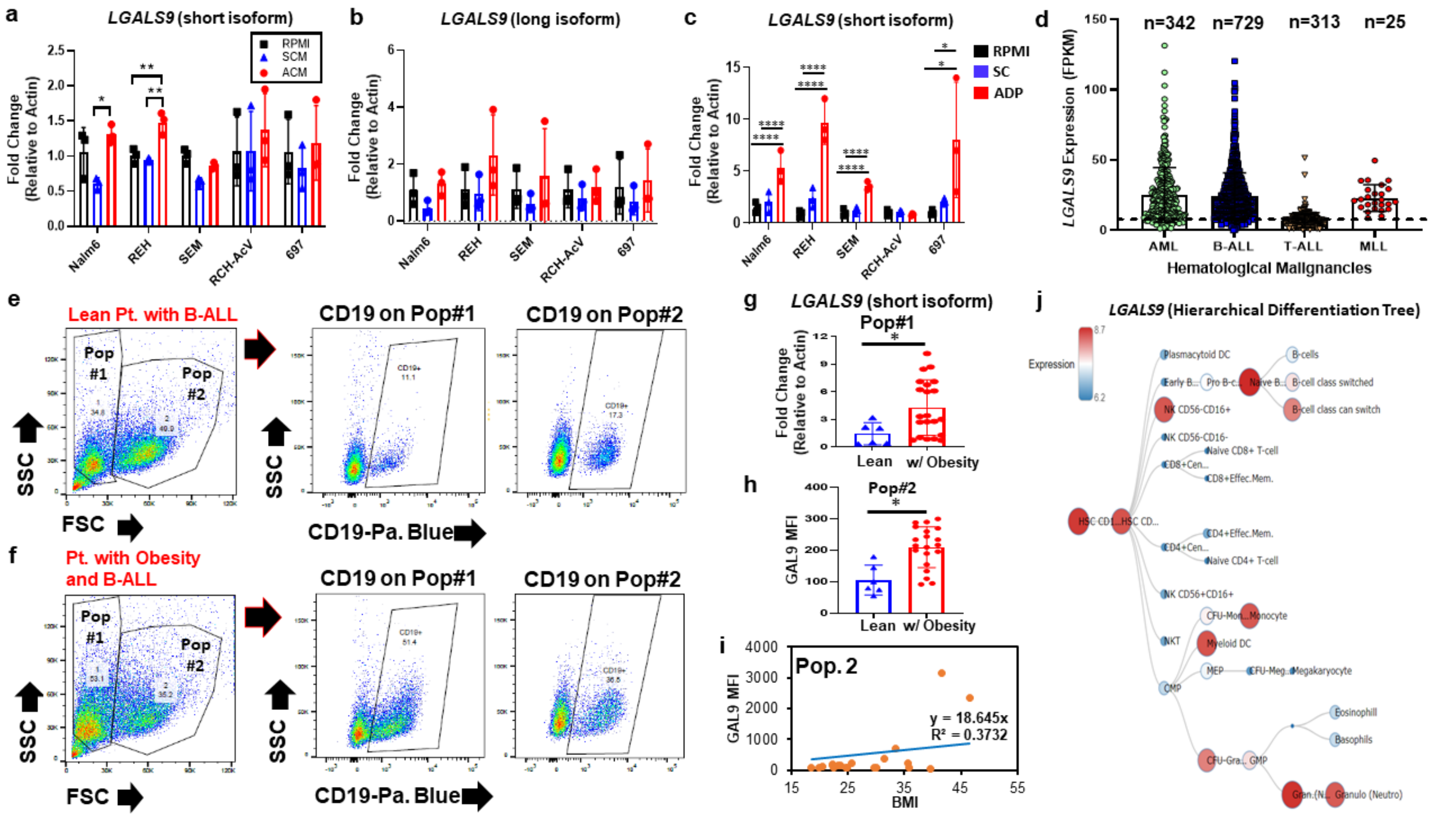

Supplementary Fig. 7. Galectin-9 is significantly upregulated in human B-ALL cells. a,b, Human B-ALL cell lines (Nalm6, REH, SEM, RCH-AcV, and 697) were cultured in unconditioned medium (RPMI), SCM, or ACM for 24 hours, RNA was harvested, and qPCR analyses were performed to determine the gene expression levels of *LGALS9*. c, Human B-ALL cells were cultured alone (RPMI) or with bone marrow stromal cells (SC) or adipocytes (ADP) for 72 hours and gene expression levels of *LGALS9* were determined using qPCR. d, *LGALS9* gene expression levels in diagnostic primary leukemia patient samples. The dotted lines show expression levels of the respective genes in PBMCs from lean individuals defined using the EMBL-EBI Expression Atlas (Genotype-Tissue Expression [GTEx] Project; n=454). Means $\pm$ s.d. are shown. e-f, The gating scheme used for flow cytometric analysis of B-ALL samples in peripheral blood isolated from lean patients and patients with obesity. B-ALL cells can express varying levels of CD19 (MFIs: Lean Pop. 1=75; Lean Pop. 2=216; With Obesity Pop. 1=145; and With Obesity Pop. 2=367). GAL-9 surface expression was assessed on cells expressing both low (population 1, Pop #1) and high (population 2, Pop #2) levels of CD19. The percentage of B-ALL blasts was greater than 80% in all patient samples at the time of collection. g, Expression of the short isoform of *LGALS9* in B-ALL cells isolated from lean and donors with obesity (w/ obesity) was determined by qPCR. h, The GAL-9 surface expression on B-ALL cells isolated from lean and donors with obesity (w/ obesity) was determined by flow cytometric analyses. i, The relationship between BMI and surface GAL-9 expression on $CD19^+$ cells (normal cells and leukemia blasts) was determined for Pop. 2. j, The BloodSpot database was mined to determine *LGALS9* gene expression levels in hematopoietic stem and progenitor cells as well as mature immune cells. The gene expression profiles of 211 samples were used to create the figure present in j. Means $\pm$ s.d. are shown in a-c and g,h (**p*<0.05, ***p*<0.01, ****p*<0.001, and *****p*<0.0001, n=3 independent experiments, one-way ANOVA with Tukey's post-test for a-c and two-sided Student's t-test for g,h).

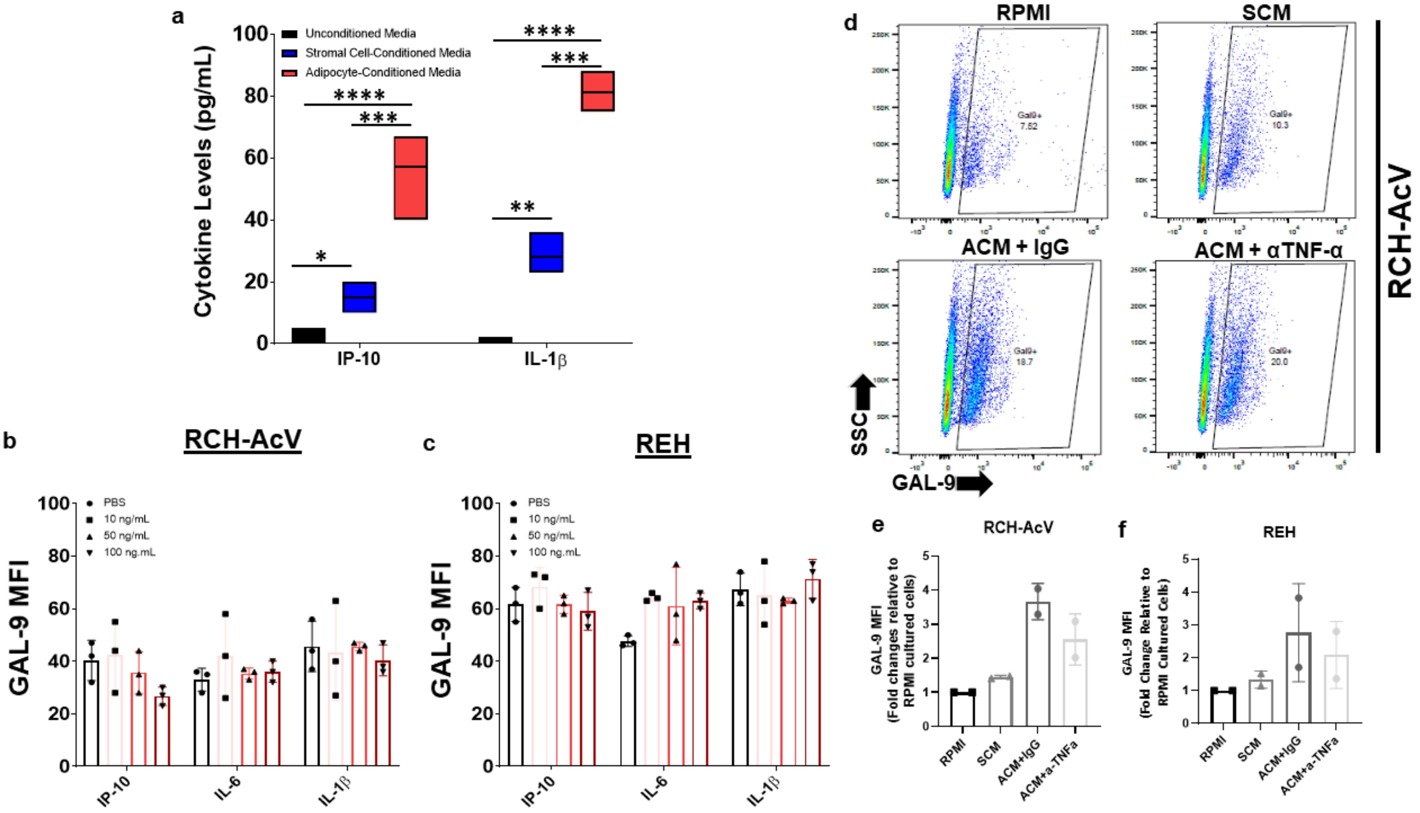

Supplementary Fig. 8. Galectin-9 surface expression on human B-ALL cells is not induced by IP-10, IL-6, and IL-1β. a, IP-10 and IL-1β levels were determined in unconditioned (RPMI), stromal-cell conditioned (SCM), and adipocyte-conditioned media (ACM) using ELISA analysis (24-hour cultures were assessed). Boxplots display the median, 25th, and 75th percentile. b-c, Human B-ALL cell lines (RCH-AcV, and REH) were cultured in unconditioned medium supplemented with PBS/control Ig, IP-10, IL-6, and IL-1β at increasing concentrations. The surface expression of GAL-9 on human B-ALL cells was determined on Day 3 of culture using flow cytometric analysis. d-f, Human B-ALL cell lines (RCH-AcV, and REH) were cultured in RPMI, SCM, and ACM (± TNF-α neutralizing antibodies) for 3 days and the surface expression of GAL-9 was determined via flow cytometry analysis. Representative primary data for RCH-AcV cells are shown in d. Means ± s.d. are shown in a-c and e,f. *p<0.05, **p<0.01, ***p<0.001, and ****p<0.0001, n=3 independent experiments/each, one-way ANOVA with Tukey's post-test.

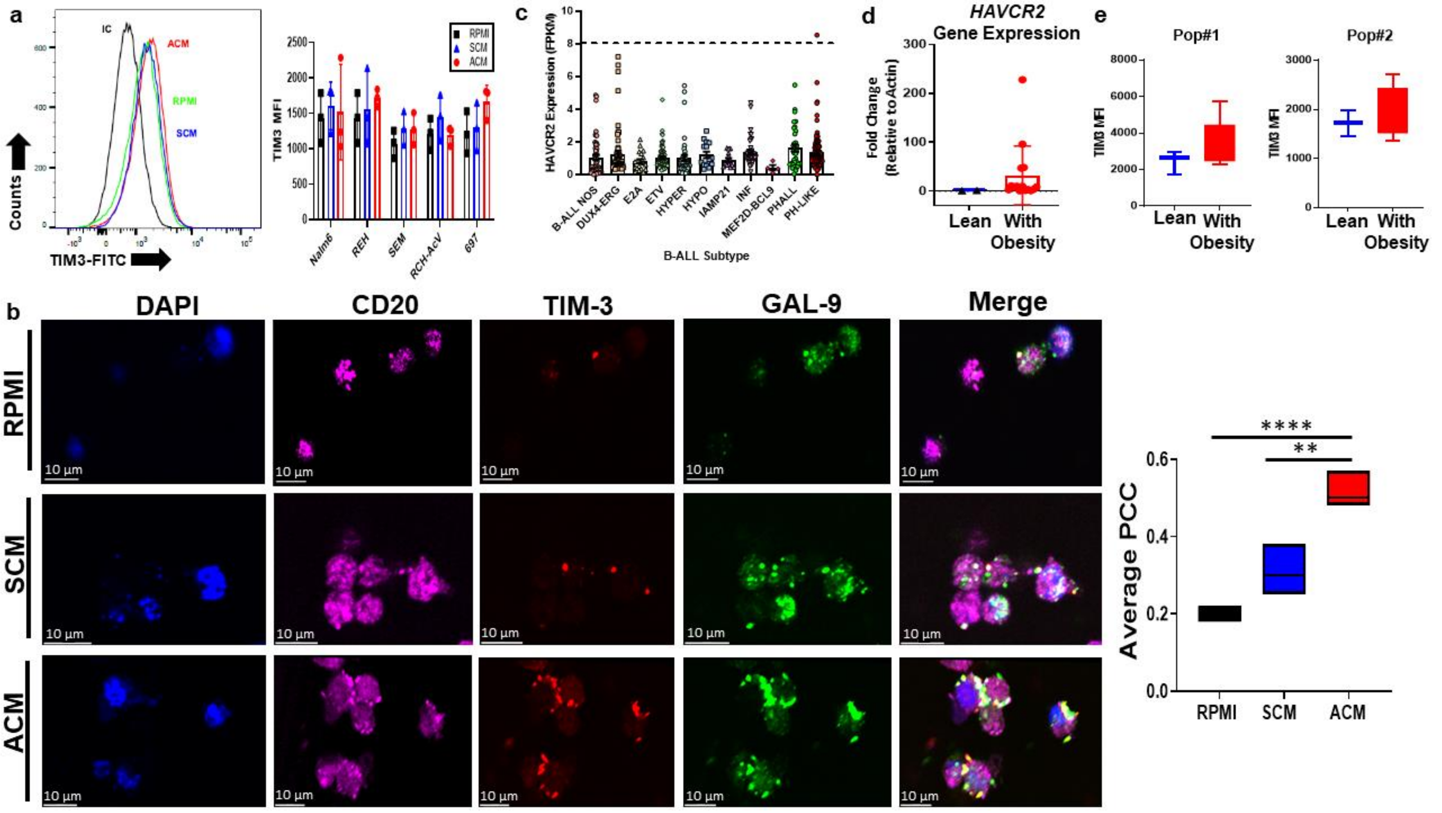

Supplementary Fig. 9. TIM-3 levels are not changed in B-ALL cells exposed to adipose-rich microenvironments but co-localization with GAL-9 is increased. a, Human B-ALL cell lines (Nalm6, REH, SEM, RCH-AcV, and 697) were cultured alone (RPMI), in bone marrow stromal cell-conditioned medium (SCM), or adipocyte-conditioned medium (ACM) for 24 hours and surface levels of TIM-3 were determined using flow cytometric analysis. b, Colocalization of GAL-9 and TIM-3 on the surface of B-ALL cells was determined via confocal microscopy after 24 hours of culture. Representative images and Pearson Correlation Coefficients (PCCs) from 697 cell cultures are shown. Boxplots display the median, 25th, and 75th percentile. c, *HAVCR2* (the gene encoding TIM-3) expression levels in samples from patients with the indicated B-ALL subtypes were determined using the St. Jude PeCan Data Portal. The dotted lines indicate expression levels in PBMCs from lean donors obtained from the EMBL-EBI Expression Atlas (Genotype-Tissue Expression [GTEx] Project; n=454). Means ± s.d. are shown. d,e *HAVCR2* gene expression levels in peripheral blood samples from lean patients and patients with obesity with B-ALL were quantified via qPCR and surface expression of TIM-3 was determined using flow cytometric analysis. In e, boxplots display the 25th and 75th percentile. In a,d, and e means ± s.d. are shown, n=3 independent experiments. **p*<0.05, ***p*<0.01, ****p*<0.001, and *****p*<0.0001, n=3 independent experiments/each, one-way ANOVA with Tukey's post-test was used to determine significance in b.

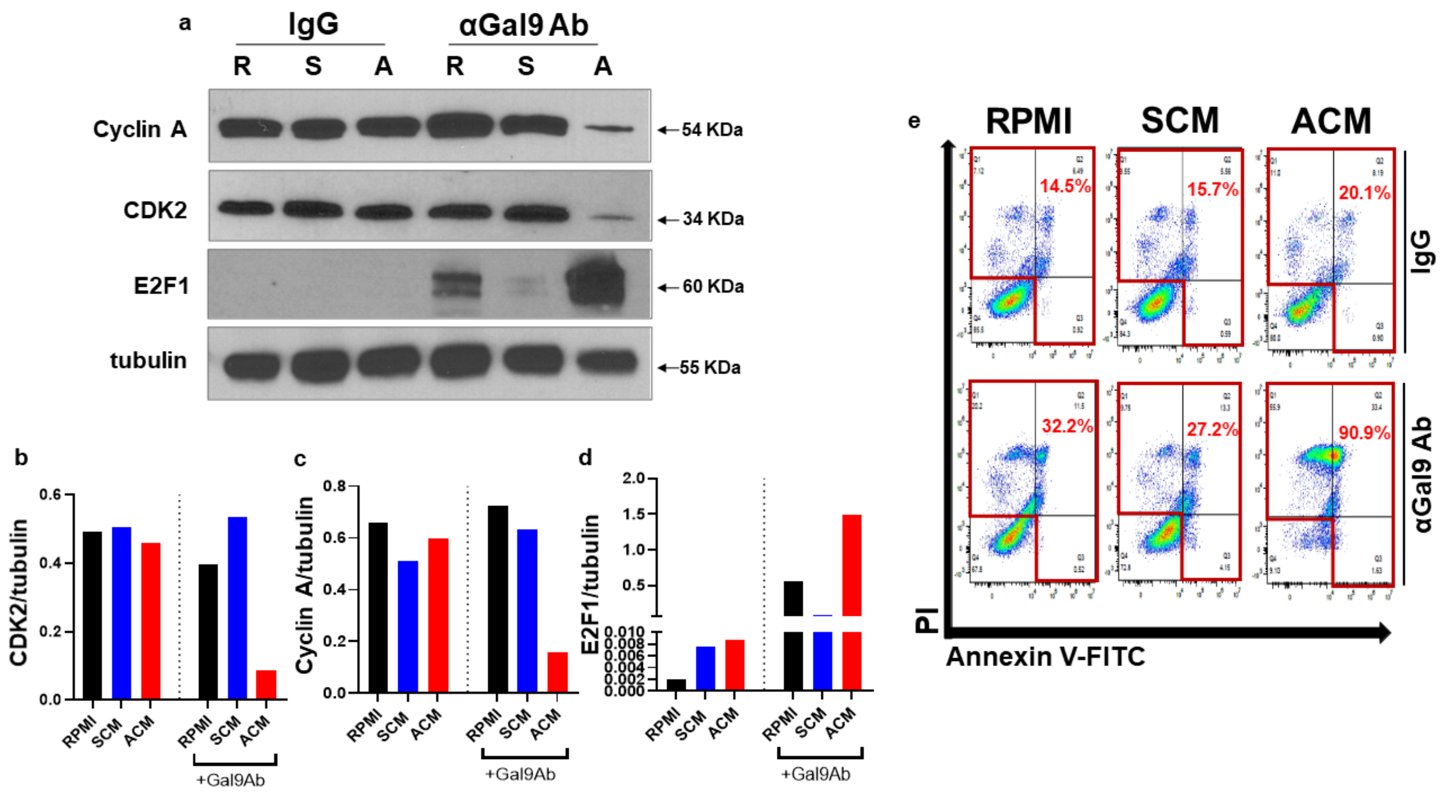

Supplementary Fig. 10. Antibody-mediated Galectin-9 inhibition on adipocyte-exposed human B-ALL cells deregulates proteins involved in cell cycle progression and is cytotoxic to human B-ALL cells. a-d, RCH-AcV cells were cultured in unconditioned medium (RPMI), stromal cell-conditioned (SCM), and adipocyte-conditioned medium (ACM) for 24 hours in the absence and presence of αGAL-9 antibody treatment. Proteins involved in cell cycle progression were assessed via western blot analysis. e, Cell death in parallel cultures was determined via Annexin-V/PI after 3 days of treatment using flow cytometry analysis. Representative survival data is shown in e. Primary western blot data shown in 'a' is representative of 4 independent experiments. Western blot source data are provided in the Source Data file.

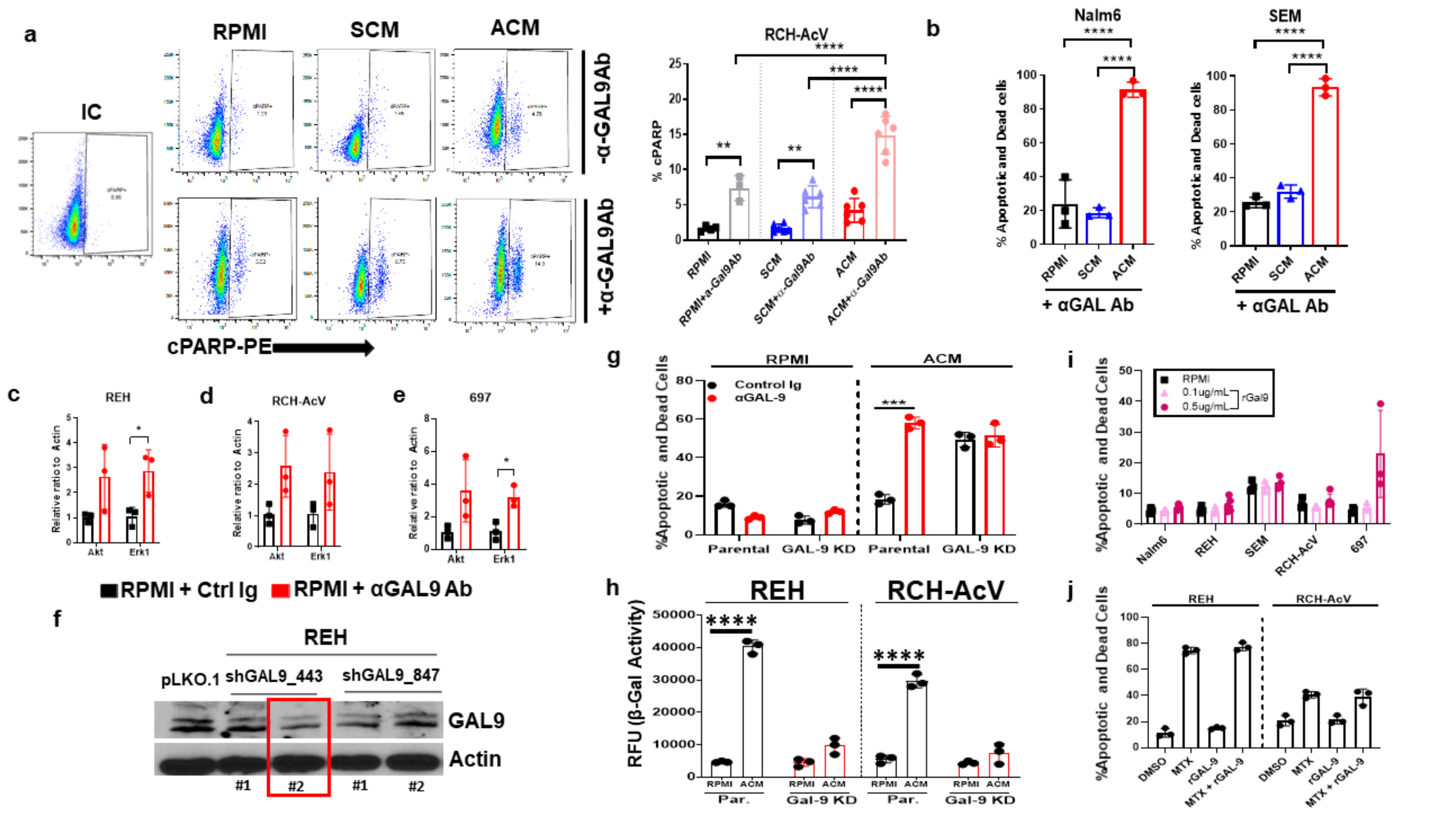

Supplementary Fig. 11. Treatment with αGalectin-9 antibody induces DNA damage and apoptosis in Human B-ALL cells. a, Human B-ALL cells were cultured for 24 hours in unconditioned medium (RPMI), stromal cell-conditioned (SCM), and adipocyte-conditioned medium (ACM) and treated with αGalectin-9 antibody (αGAL-9 Ab) or IgG control (IgG ctrl) for an additional 2 days. Cleaved PARP activation, a DNA damage indicator, was determined. b, Human B-ALL cell lines (Nalm6 and SEM) were cultured for 24 hours in RPMI, SCM, or ACM and then treated with αGAL-9 Ab or IgG ctrl for an additional 2 days. The percentage of apoptotic and dead cells on day 3 of culture was determined using Annexin-V/ PI staining. c-e, Human B-ALL cells were cultured in RPMI + Ctrl Ig or RPMI + αGAL-9 Ab for 48 hours, RNA was isolated, and qPCR analysis was performed to determine *AKT* and *ERK1* gene expression levels. f-g, GAL-9 was knocked down in REH cells using shRNA methodology, and construct #443 exhibiting reduced protein expression (western blot analysis). Sample preparation number 2 (indicated in the red box) was cultured in RPMI or ACM and treated with Ctrl Ig or αGAL-9 Ab. The percentage of apoptotic and dead cells on day 3 of culture was determined using Annexin-V/ PI staining followed by flow cytometric analysis. h, Human B-ALL cells (parental and GAL-9 knockdown) were cultured in RPMI or ACM for 24 hours. Lysates were harvested and β-Galactosidase activity was determined using the Senescence β-Galactosidase Activity Assay Kit (Cell Signaling Technology). i,j, Human B-ALL cells were cultured in RPMI and treated with Ctrl Ig (RPMI group) or rGAL-9 for 3 days in the absence (i) and presence (j) of MTX. The percentage of apoptotic and dead cells on day 3 of culture was determined as described above. Means ± s.d. are shown (**p*<0.05, ***p*<0.01, ****p*<0.001, *****p*<0.0001). For a-e and 10g-j, each dot represents the average of technical replicates from independent experiments (n=3). One-way ANOVA with Tukey's post-test (b,g) or two-sided Student's t-test (a, c-e, h-j) were performed to determine statistical significance. Western blot source data are provided in the Source Data file.

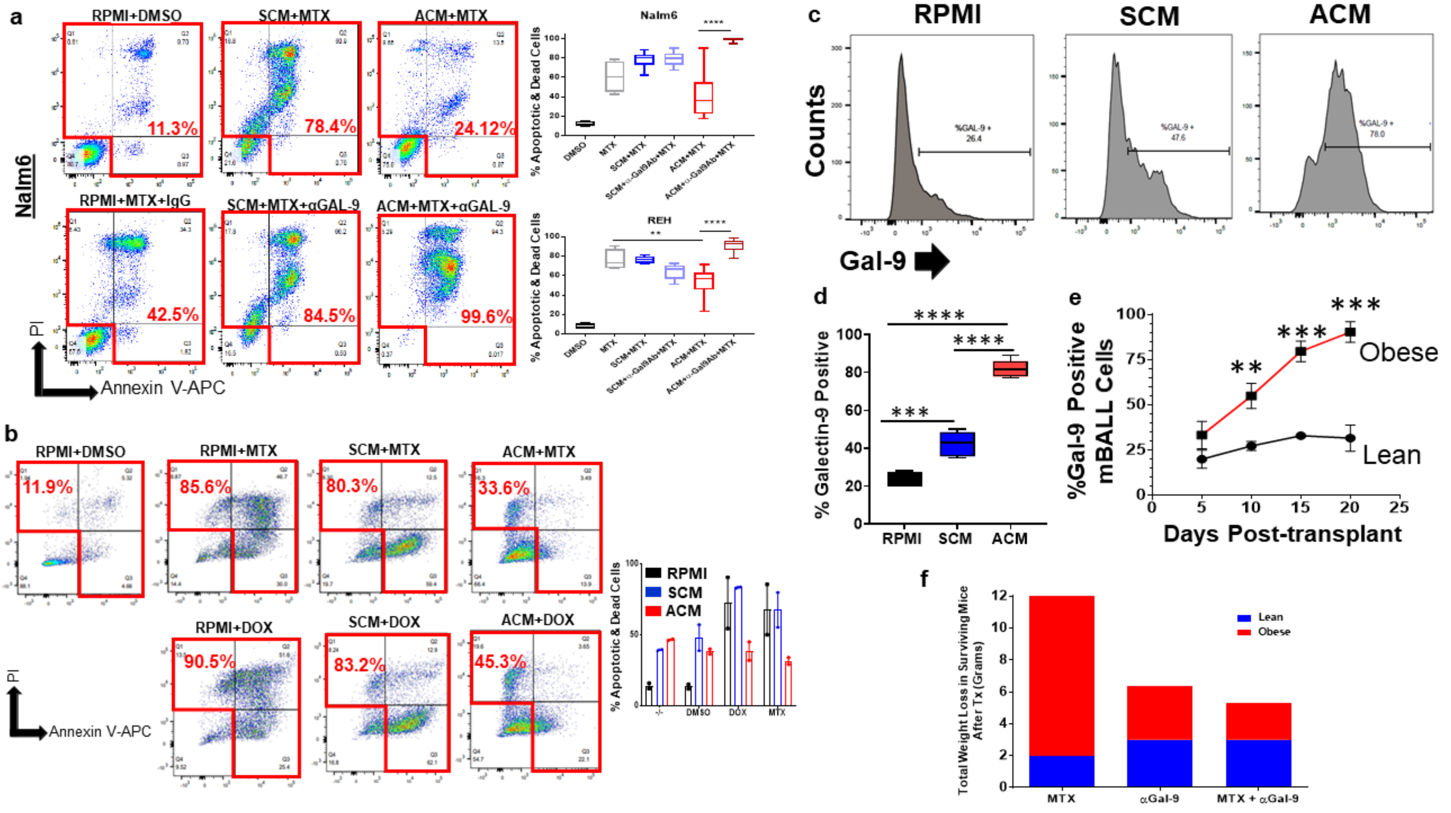

Supplementary Fig. 12. Murine B-ALL cells phenocopy human B-ALL exposed to methotrexate (MTX) and αGal-9 antibodies are well-tolerated alone and in combination with MTX. a, Human B-ALL cell lines (Nalm6 and REH) were cultured for 24 hours in unconditioned medium (RPMI), bone marrow stromal cell-conditioned medium (SCM), or adipocyte-conditioned medium (ACM) and then treated with anti-GAL-9 antibody (αGAL-9 Ab) or an IgG control antibody (IgG ctrl) for an additional 2 days with MTX. The percentage of apoptotic and dead cells on day 3 of culture was determined using Annexin-V/ PI staining followed by flow cytometric analysis. Box and Whisker plots display the median, 25th, and 75th percentile. The whiskers extend from the minima to the maxima, respectively. b, Murine Bcr-Abl+ *Arf-/-* B-ALL cells (mB-ALL) were cultured in RPMI, SCM, or ACM for 24 hours prior to treatment with methotrexate (MTX) or doxorubicin (DOX) for an additional 2 days. The percentage of apoptotic and dead cells on day 3 of culture was determined using Annexin-V/ PI staining and flow cytometric analysis. c,d, mB-ALL cells were cultured in RPMI, SCM, or ACM for 3 days, and the percentage of Gal-9 expressing cells was determined via flow cytometric analysis. Box and Whisker plots display the median, 25th, and 75th percentile. The whiskers extend from the minima to the maxima, respectively. e, 10^5 GFP-expressing mB-ALL cells were injected into wild-type mice. Mice were bled every 5 days, and the percentage of Galectin-9 expressing B-ALL was determined via flow cytometric analysis. f, Weight loss in surviving lean and obese wild-type mice after 30 days of treatment is shown. Means ± s.d. are shown in a,b and d,e with **p*<0.05, ***p*<0.01, ****p*<0.001, and *****p*<0.0001. One-way ANOVA with Tukey's post-test was used to determine statistical significance in a,d. Two-sided Student's t-test was performed relative to the lean control with values for b,e. For a (n=4), b (n=2) and d,e (n=4), data represent the average of technical replicates from each independent experiment.

Supplementary Table 1. Sequences of Primers used for Quantitative PCR

Name of Genes	Sequences	Product Size	Reference
hGal9_Forward	5'-CAGTGCTCAGAGGTTCCACA-3'		
hGal9_Reverse	5'-TGAGGCAGTGAGCTTCACAC-3'	206bp	[1]
hGal9L_Forward	5'-CTTCCACCGCGTGCCCTTCC-3'		
hGal9L_Reverse	5'-TTTTGTCTGCGCCCCCTGGG-3'	176bp	[1]
hTIM3_Forward	5'-TCCAAGGATGCTTACCACCAG-3		
hTIM3_Reverse	5'-GCCAATGTGGATATTTGTGTTAGA TT-3'	96bp	[2]
hAkt1_Forward	5'-TCTATGGCGCTGAGATTGTG-3'		
hAkt1_Reverse	5'-CTTAATGTGCCCGTCCTTGT-3'	113bp	[3]
hADGL1_Forward	5'-CAGTACGACTGTGTCCCCTACA-3'		
hADGL1_Reverse	5'-GCACCATGCGCCAGACTG-3'	123bp	[4]
hErk1_Forward	5'-CCTGCGACCTTAAGATTTGTGATT-3'		
hErk1_Reverse	5'-CAGGGAAGATGGGCCGGTTAGAGA-3'	210bp	[5]
hErk2_Forward	5'-CGTGTTGCAGATCCAGACCATGAT-3'		
hErk2_Reverse	5'-TGGACTTGGTGTAGCCCTTGGAA-3'	112bp	[6]
hNF-κB_Forward	5'-ATGGCTTCTATGAGGCTGAG-3'		
hNF- κB_Reverse	5'-GTTGTTGTTGGTCTGGATGC-3'	128bp	[7]
hSTAT3_Forward	5'-GAGAAGGACATCAGCGGTAAG-3'		
hSTAT3_Reverse	5'-AGTGGAGACACCAGGATATTG-3'	137bp	[8]
hSTAT5A_Forward	5'-TTACTGAAGATCAAGCTGGGG-3'		
hSTAT5A_Reverse	5'-TCATTGTACAGAATGTGCCGG-3'	104bp	[9]
hGAPDH_Forward	5'-AGGGCTGCTTTTAACTCTGGTAAA-3		
hGAPDH_Reverse	5'-CATATTGGAACATGTAAACCATGTAGT TG-3	91bp	[2]

Gene	Sequences	Size (bp)	Ref
DYRK4	F_5'-GCTGTTGAAAGCCTGCAGC-3'<br>R_5'-GACTTCTCCTCTGCCTTGGG-3'	236	[10]
EGR1	F_5'-CTTCAACCCTCAGGCGGACA-3'<br>R_5'-GGAAAAGCGGCCAGTATAGGT-3'	160	[11]
FAM45A	F_5'-AAGAATTGTGGTGTATCACCCC-3'<br>R_5'-CAAATCCAGCGACGTAACCTG-3'	170	[12]
HECTD4	F_5'-TGTAGTGCTGGAGAGCGACTTG-3'<br>R_5'-CTGTCTTCGGTCAGCCTGCAAA-3'	114	Origene, HECTD4 Human qPCR Primer Pair,(NM_001109662)
LIG1	F_5'-AGATCGAGGAGGTGTCTGCT-3'<br>R_5'-AGGCTGAGGTAGAGGACAGG-3'	112	
LRPAP1	F_5'-TCACGAGTACAACGTCCTGC-3'<br>R_5'-GGGGCTAATGACGTTCTCGT-3'	70	
LSM8	F_5'-TCAGCTCTTCACAGGGGGTA-3'<br>R_5'-ACTGCAACGTTGTCACCTCT-3'	70	
NFATC4	F_5'-CATCCTACAGACCGGGCCT-3'<br>R_5'-CCTGTGGTACCCCTAGTCTCAGG-3'	52	[13]
PRR14	F_5'-GCACCACAGCTACCATCAGG-3'<br>R_5'-CCGGTCCACCTTTTGTGAAG-3'	211	[14]
PVT1	F_5'-CTTTCAGCACTCTGGACGGA-3'<br>R_5'-ACACAGAGCACCAAGACTGG-3'	100	
RCN1	F_5'-TGCAGACCTCAATGGTGACCT-3'<br>R_5'-AAGGCAGTGAACTCCTCCCG-3'	51	[15]
TMEM8B	F_5'-TTAATGTCCGTGTGGGTCAC-3'<br>R_5'-CAGAGCCATGGACAGCAGCA-3'	70	[16]
TRPM2	F_5'-ACGTGCTCATGGTGGACTTC-3'<br>R_5'-AGGGTCATAGAAGAGCTGCC-3'	110	[17]
TSEN2	F_5'-CGCTCTTCCGAAAGTCCTCC-3'<br>R_5'-GGGGCATGGAAAACTGCTTC-3'	87	
ZBTB20	F_5'-ATGCTAGAACGGAAGAAACCCA-3'<br>R_5'-TGTGAGCGTGAGAGTTTGTCA-3'	184	[18]
Zfp36L2	F_5'-GATGTCGACTTCTTGTGCAAGACA-3'<br>R_5'-GCGTCCCCACCGCCTTCT-3'	85	[19]
ZGLP1	F_5'-GAGACGCTGAAGATGGGACC-3'<br>R_5'-CATAGCCTCTTGGGCTGGAC-3'	121	

Note: Quantitative PCR experiments were performed to validate specific targets nominated in our RNA-sequencing analysis. A few of the validated targets are highlighted in this manuscript.

Supplementary References

1. Gieseke, F., et al., *Proinflammatory stimuli induce galectin-9 in human mesenchymal stromal cells to suppress T-cell proliferation.* Eur J Immunol, 2013. **43**(10): p. 2741-9.
2. Khademi, M., et al., *T Cell Ig- and mucin-domain-containing molecule-3 (TIM-3) and TIM-1 molecules are differentially expressed on human Th1 and Th2 cells and in cerebrospinal fluid-derived mononuclear cells in multiple sclerosis.* J Immunol, 2004. **172**(11): p. 7169-76.
3. Zhang, J., et al., *Regulation of AKT gene expression by cisplatin.* Oncol Lett, 2013. **5**(3): p. 756-760.
4. Boucard, A.A., S. Maxeiner, and T.C. Sudhof, *Latrophilins function as heterophilic cell-adhesion molecules by binding to teneurins: regulation by alternative splicing.* J Biol Chem, 2014. **289**(1): p. 387-402.
5. Wang, X., et al., *Effects and relationship of ERK1 and ERK2 in interleukin-1beta-induced alterations in MMP3, MMP13, type II collagen and aggrecan expression in human chondrocytes.* Int J Mol Med, 2011. **27**(4): p. 583-9.
6. Shan, J., et al., *MAPK signaling triggers transcriptional induction of cFOS during amino acid limitation of HepG2 cells.* Biochim Biophys Acta, 2015. **1853**(3): p. 539-48.
7. Jiang, Z.L., et al., *Hypoxia regulates iNOS expression in human normal peritoneal and adhesion fibroblasts through nuclear factor kappa B activation mechanism.* Fertil Steril, 2009. **91**(2): p. 616-21.
8. Turton, K.B., et al., *Ratios of Four STAT3 Splice Variants in Human Eosinophils and Diffuse Large B Cell Lymphoma Cells.* PLoS One, 2015. **10**(5): p. e0127243.
9. Vieyra-Garcia, P.A., et al., *STAT3/5-Dependent IL9 Overexpression Contributes to Neoplastic Cell Survival in Mycosis Fungoides.* Clin Cancer Res, 2016. **22**(13): p. 3328-39.
10. Papadopoulos, C., et al., *Splice variants of the dual specificity tyrosine phosphorylation-regulated kinase 4 (DYRK4) differ in their subcellular localization and catalytic activity.* J Biol Chem, 2011. **286**(7): p. 5494-505.
11. Demaria, S., et al., *Cancer and inflammation: promise for biologic therapy.* J Immunother, 2010. **33**(4): p. 335-51.
12. Cheng, J., et al., *Understanding the Mechanisms of Resistance to CAR T-Cell Therapy in Malignancies.* Front Oncol, 2019. **9**: p. 1237.
13. Perroud, J., et al., *Distinct roles of NFATc1 and NFATc4 in human primary myoblast differentiation and in the maintenance of reserve cells.* J Cell Sci, 2017. **130**(18): p. 3083-3093.
14. Aleman, F.D.D. and D.R. Valenzano, *Microbiome evolution during host aging.* PLoS Pathog, 2019. **15**(7): p. e1007727.
15. Cooper, C.R., et al., *Novel surface expression of reticulocalbin 1 on bone endothelial cells and human prostate cancer cells is regulated by TNF-alpha.* J Cell Biochem, 2008. **104**(6): p. 2298-309.
16. Millay, D.P., et al., *Structure-function analysis of myomaker domains required for myoblast fusion.* Proc Natl Acad Sci U S A, 2016. **113**(8): p. 2116-21.
17. Braig, M., et al., *A 'telomere-associated secretory phenotype' cooperates with BCR-ABL to drive malignant proliferation of leukemic cells.* Leukemia, 2014. **28**(10): p. 2028-39.
18. Chiong, M., et al., *Cardiomyocyte death: mechanisms and translational implications.* Cell Death Dis, 2011. **2**: p. e244.
19. Behan, J.W., et al., *Activation of adipose tissue macrophages in obese mice does not require lymphocytes.* Obesity (Silver Spring), 2013. **21**(7): p. 1380-8.
