# Supplementary material for: Obesity-induced galectin-9 is a therapeutic target in B-cell acute lymphoblastic leukemia
Source: Nat Commun. 2022 Mar 3;13:1157. doi: 10.1038/s41467-022-28839-y (PMC8894417; doi:10.1038/s41467-022-28839-y)
Supplement: Supplementary file 3 — Reporting summary [file 41467_2022_28839_MOESM3_ESM.pdf]

nature research

Corresponding author(s): Curtis J. Henry

Last updated by author(s): Jan 8, 2022

Reporting Summary

Nature Research wishes to improve the reproducibility of the work that we publish. This form provides structure for consistency and transparency in reporting. For further information on Nature Research policies, see Authors & Referees and the Editorial Policy Checklist .

Statistics

For all statistical analyses, confirm that the following items are present in the figure legend, table legend, main text, or Methods section.

n/a	Confirmed	
☐	☒	The exact sample size (*n*) for each experimental group/condition, given as a discrete number and unit of measurement
☐	☒	A statement on whether measurements were taken from distinct samples or whether the same sample was measured repeatedly
☐	☒	The statistical test(s) used AND whether they are one- or two-sided<br>*Only common tests should be described solely by name; describe more complex techniques in the Methods section.*
☐	☒	A description of all covariates tested
☐	☒	A description of any assumptions or corrections, such as tests of normality and adjustment for multiple comparisons
☐	☒	A full description of the statistical parameters including central tendency (e.g. means) or other basic estimates (e.g. regression coefficient) AND variation (e.g. standard deviation) or associated estimates of uncertainty (e.g. confidence intervals)
☐	☒	For null hypothesis testing, the test statistic (e.g. *F*, *t*, *r*) with confidence intervals, effect sizes, degrees of freedom and *P* value noted<br>*Give P values as exact values whenever suitable.*
☒	☐	For Bayesian analysis, information on the choice of priors and Markov chain Monte Carlo settings
☐	☒	For hierarchical and complex designs, identification of the appropriate level for tests and full reporting of outcomes
☐	☒	Estimates of effect sizes (e.g. Cohen's *d*, Pearson's *r*), indicating how they were calculated

Our web collection on statistics for biologists contains articles on many of the points above.

Software and code

Policy information about availability of computer code

Data collection	The software programs used for data analysis are listed below. No software was used for data collection.
Data analysis	1. Survival Analysis of primary patient gene expression data was performed using CASAS software (Rupji, M., X. Zhang, and J. Kowalski, CASAS: Cancer Survival Analysis Suite, a web based application. F1000Res, 2017. 6: p. 919; Version 2).<br><br>2. All flow samples were analyzed by using Flowjo Portal Software Version 9 (Ashland, Oregon).<br><br>3. GraphPad Prism (San Diego, California) Software Version 9.1.1 was used to determine statistical significance for the data presented in this manuscript.<br><br>4. Image J (Version 1.53n) was used for all microscopy experiments presented in this manuscript.<br><br>5. BioRender.com (Toronto, Ontario) was used to create all model images used in this manuscript.

For manuscripts utilizing custom algorithms or software that are central to the research but not yet described in published literature, software must be made available to editors/reviewers. We strongly encourage code deposition in a community repository (e.g. GitHub). See the Nature Research guidelines for submitting code & software for further information.

Data

Policy information about availability of data

All manuscripts must include a data availability statement. This statement should provide the following information, where applicable:

- Accession codes, unique identifiers, or web links for publicly available datasets
- A list of figures that have associated raw data
- A description of any restrictions on data availability

1. All clinical and mRNA-seq expression data for Acute Lymphoblastic Leukemia (ALL) Phase II project was downloaded from the TARGET Data Matrix Portal (TARGET Data Matrix Portal: https://ocg.cancer.gov/programs/target/data-matrix; NIH and NCI; accessed in June 2017).

2. Patient derived LGALS9 (GAL-9) and HAVCR2 (TIM-3) gene expression levels were determined using the St. Jude Cloud Pecan Bioportal (https://pecan.stjude.cloud/).

3. The expression of LGALS9 and HAVCR2 in normal B-cells was determined using The Genotype-Tissue Expression (GTEx) project - GTEx Portal (https://www.gtexportal.org/home/).

4. The datasets generated during and/or analyzed in the current study are available from the corresponding author on reasonable request (for Table 1 and Figure 4). The raw data and processed RNA-seq data used in this study are deposited in the GEO repository under accession number GSE183062 and can be accessed without restrictions. Source data are provided with this paper.

Field-specific reporting

Please select the one below that is the best fit for your research. If you are not sure, read the appropriate sections before making your selection.

☒ Life sciences ☐ Behavioural & social sciences ☐ Ecological, evolutionary & environmental sciences

For a reference copy of the document with all sections, see nature.com/documents/nr-reporting-summary-flat.pdf

Life sciences study design

All studies must disclose on these points even when the disclosure is negative.

Sample size	For RNA-seq experiments, sample size was not determined. For mouse experiments, power analysis was performed based on preliminary estimates of leukemia burden and variance. The number of animals per group was chosen so as to provide more than 90% statistical power at an alpha levelof 0.05 to detect the difference between treatment groups. All tissue culture experiments were performed in triplicates to allow for calculation of standard deviation, standard error of the mean and t-statistics for use in two-tailed Student's t-test or ANOVA.
Data exclusions	No data was excluded from the analysis.
Replication	All experiments were performed in triplicates with some experiments performed with greater than 10 independent experiments. We did not exclude any replicates. We performed one experiment in human B-ALL cells treated with DMSO or MTX for the RNA-sequencing studies presented in this manuscript. Information pertaining to the number of independent replications for each experiment are included in the figure legends.
Randomization	Both male and female mice were used in the studies presented in this manuscript. Mice were randomized based on diet and treatment groups (vehicle control, MTX, and anti-Galectin-9). Using both male and female mice in these studies ensured that sex as a biological variable was addressed for leukemia progression, as well as, treatment responses. All other experiments were randomized using randomly assigned plates of cells per condition.
Blinding	For survival experiments, mice were removed for these studies at the first sign of morbidity. The treatment groups were unknown to the investigator removing mice for euthanasia due to signs of illness or distress; thereby, avoiding bias in these experiments. For all microscope images, slides were double blinded prior to analysis. For all other experiments, sample were harvested and processed by scientists without knowledge of hypothesized treatment effects. After processing, results were properly labeled once data processing had been completed.

Reporting for specific materials, systems and methods

We require information from authors about some types of materials, experimental systems and methods used in many studies. Here, indicate whether each material, system or method listed is relevant to your study. If you are not sure if a list item applies to your research, read the appropriate section before selecting a response.

Materials & experimental systems

n/a	Involved in the study	
☐	☒	Antibodies
☐	☒	Eukaryotic cell lines
☒	☐	Palaeontology
☐	☒	Animals and other organisms
☐	☒	Human research participants
☒	☐	Clinical data

Methods

n/a	Involved in the study	
☒	☐	ChIP-seq
☐	☒	Flow cytometry
☒	☐	MRI-based neuroimaging

Antibodies

Antibodies used

Western Blots:
Anti-Fatty acid binding protein-4 (cat# AF1443, R&D Systems); 1:4000
Anti-goat HRP (cat# AP180p, EMD Millipore); 1:10000
Anti-GAL9 (cat# ab69630, Abcam); 1:500
Anti-TIM3 (cat# ab47997, Abcam); 1:1000
Anti-β-actin (cat# 4967, Cell Signaling); 1:5000
Anti-CDK4 (cat# 12790s, Cell Signaling); 1:500
Anti-Cyclin D3 (cat# 2936s, Cell Signaling); 1:500
Anti-Cyclin A (cat# sc-271682, Santa Cruz Biotechnology); 1:500
Anti-Cyclin D2 (cat# 2978S, Cell Signaling Technology); 1:500
Anti-E2F1 (cat# sc-56661, Santa Cruz Biotechnology); 1:500
Anti-γH2AX (cat# 2577s, Cell Signaling); 1:1000
Anti-Caspase 3 (cat# 9664s and 9661s, Cell Signaling Technology); 1:1000/each
Anti-BCL-xL (cat# 2764S, Cell Signaling Technology); 1:500
Anti-BIM (cat# 2933S, Cell Signaling Technology); 1:500
Anti-BAX (cat# 5023S, Cell Signaling Technology); 1:500
Anti-X-linked inhibitor of apoptosis (XIAP; cat# 2042S, Cell Signaling Technology); 1:500
Anti-ATR (cat# 2790S, Cell Signaling Technology); 1:500
Anti-cRAF (cat# 9422S, Cell Signaling Technology); 1:500
Anti-pERK (cat# 9101S, Cell Signaling Technology); 1:500
Anti-ERK (cat# 9102S, Cell Signaling Technology); 1:500
Anti-pAKT (cat# 9271S, Cell Signaling Technology); 1:500
Anti-AKT (cat# 4691S, Cell Signaling Technology); 1:500
β-tubulin (cat# 2144S, Cell Signaling Technology); 1:5000
Anti-mouse HRP (cat# AP308P, EMD Milipore); 1:10000
Anti-rabbit HRP (cat# 401315, Calbiochem); 1:10000

Flow Cytometry:
Anti-GAL9 (cat#348908, BioLegend); 1:200
Anti-TIM3 (cat#130-126-004, Miltenyi Biotec); 1:200

Confocal Microscopy:
Anti-GAL9 (cat# ab69630, Abcam); 1:500
Anti-TIM-3 (cat# ab47997, Abcam); 1:1000
Anti-CD20 Ab-1 (cat# MS-340-S0, Thermo Fisher Scientific); 1:200
Goat anti-Rabbit IgG (H +L) Highly Cross-Absorbed Secondary Antibody AlexaFluor-488 (cat# A11034, Invitrogen); 1:100
Donkey anti-Goat IgG (H + L) Cross-Absorbed Secondary Antibody AlexaFluor-568 (cat# A11057, Invitrogen); 1:100
Goat anti-Mouse IgG (H + L) Cross-Absorbed Secondary AntibodyAlexaFluor-635 (cat# A31574, Invitrogen); 1:100

Validation

Anti-Fatty acid binding protein-4 (cat# AF1443, R&D Systems). 16 citations for validation on the manufacture's website. Detects mouse FABP4 in direct ELISAs and Western Blots. Less than 5% cross-reactivity with recombinant mouse FABP9 and FABP5 observed. As reported on the manufacturer's website, a single band of 14kDA is observed in mouse thymus and heart tissues. Similar results were obtained in our studies for adipocytes.

Anti-GAL9 (cat# ab69630, Abcam). 20 citations for validation on the manufacture's website. Validated in ELISA, IHC-P, western blot, and ICC/IF experiments. Reacts with mouse, rat, and human GAL-9. Website provided ICC/IF validation in NIH/3T3 cells (1:500 antibody dilution). Similar results were obtained in our studies with human B-ALL cells.

Anti-TIM3 (cat# ab47997, Abcam). 6 citations for validation on the manufacture's website. Validated for western blot in Jurkat and MOLT4 cells; whereas, A431 cells were used as a negative control. Reacts with human TIM3. Similar results were obtained in our studies with human B-ALL cells.

Anti-β-actin (cat# 4967, Cell Signaling). 2042 citations for validation on the manufacture's website. Validated for western blot in HeLa, C2C12, C6, COS, and MvLu cells. Molecular weight is 45 kDa and reactive with human, mouse, rat, hamster, monkey, mink, D. melanogaster, zebrafish, and bovine. Similar results were obtained in our studies.

Anti-CDK4 (cat# 12790s, Cell Signaling). 259 citations for validation on the manufacture's website. Validated for western blot in Jurkat, HeLa, MCF7, and COS-7 cells. Molecular weight is 30 kDa and reactive with human and monkey. Similar results were obtained in our studies.

Anti-Cyclin D3 (cat# 2936s, Cell Signaling). 186 citations for validation on the manufacture's website. Validated for western blot in SK-N-MC, C6, and IMCD3 cells. Molecular weight is 31 kDa and reactive with human, mouse, and rat. Similar results were obtained in our studies.

Anti-Cyclin D2 (cat# 2978S, Cell Signaling Technology). 804 citations for validation on the manufacture's website. Validated for western blot in MCF7, L929, and C6 cells. Molecular weight is 36 kDa and reactive with human, mouse, and rat. Similar results were obtained in our studies.

Anti-γH2AX (cat# 2577s, Cell Signaling). 620 citations for validation on the manufacture's website. Validated for western blot in untreated or UV-treated 293 cells. Molecular weight is 15 kDa and reactive with human, mouse, rat, and monkey. Similar results were obtained in our studies.

Anti-Caspase 3 (cat# 9664s and 9661s, Cell Signaling Technology). Cat #9664s, 3075 citations for validation on the manufacture's website. Validated for western blot in C6 (rat), NIH/3T3 (mouse), and Jurkat (human) cells untreated or treated with staurosporine or etoposide. Molecular weight is 17, 19 kDa and reactive with human, mouse, rat, and monkey. Cat #9661s, 5672 citations for validation on the manufacture's website. Validated for western blot in HeLa, NIH/3T3, and C6 cells untreated or treated with staurosporine or cytochrome-c. Molecular weight is 17, 19 kDa and reactive with human, mouse, rat, and monkey. Similar results were obtained in our studies with both antibodies.

Anti-BCL-xL (cat# 2764S, Cell Signaling Technology). 728 citations for validation on the manufacture's website. Validated for western blot in Jurkat and HeLa (human), COS (monkey), NIH/3T3, and L929 (mouse) cells. Molecular weight is 30 kDa and reactive with human, mouse, rat, and monkey. Similar results were obtained in our studies.

Anti-BIM (cat# 2933S, Cell Signaling Technology). 340 citations for validation on the manufacture's website. Validated for western blot in Raji, A20, and RL cells. Molecular weight is 12, 15, and 23 kDa and reactive with human, mouse, and rat. Similar results were obtained in our studies.

Anti-BAX (cat# 5023S, Cell Signaling Technology). 689 citations for validation on the manufacture's website. Validated for western blot in HeLa cells. Molecular weight is 20 kDa and reactive with human. Similar results were obtained in our studies.

Anti-X-linked inhibitor of apoptosis (XIAP; cat# 2042S, Cell Signaling Technology). 119 citations for validation on the manufacture's website. Validated for western blot in HeLa, C6, BaF3 cells. Molecular weight is 53 kDa and reactive with human, mouse, rat, and monkey. Similar results were obtained in our studies.

Anti-ATR (cat# 2790S, Cell Signaling Technology). 101 citations for validation on the manufacture's website. Validated for western blot in HeLa and HT-29 cells. Molecular weight is 300 kDa and reactive with human and monkey. Similar results were obtained in our studies.

Anti-cRAF (cat# 9422S, Cell Signaling Technology). 152 citations for validation on the manufacture's website. Validated for western blot in HeLa, C2C12, and PC-12 cells. Molecular weight is 65 to 75 kDa and reactive with human, mouse, rat, and monkey. Similar results were obtained in our studies.

Anti-pERK (cat# 9101S, Cell Signaling Technology). 5554 citations for validation on the manufacture's website. Validated for western blot in MEFs treated with bFGF or PDGF. Molecular weight is 42, 44 kDa and reactive with human, mouse, rat, monkey, mink, D. melanogaster, zebrafish, bovine, pig, and C. elegans. Similar results were obtained in our studies.

Anti-ERK (cat# 9102S, Cell Signaling Technology). 4986 citations for validation on the manufacture's website. Validated for western blot in HeLa cells. Molecular weight is 42, 44 kDa and reactive with human, mouse, rat, hamster, monkey, mink, zebrafish, bovine, pig, and S. cerevisiae. Similar results were obtained in our studies.

Anti-pAKT (cat# 9271S, Cell Signaling Technology). 5197 citations for validation on the manufacture's website. Validated for western blot in NIH/3T3 cells left untreated or treated with PDGF, wortmannin, LY294002, rapamycin, or PD98059. Molecular weight is 60 kDa and reactive with human, mouse, rat, hamster, monkey, D. melanogaster, bovine, and dog. Similar results were obtained in our studies.

Anti-AKT (cat# 4691S, Cell Signaling Technology). 2681 citations for validation on the manufacture's website. Validated for western blot in various cell lines. Molecular weight is 60 kDa and reactive with human, mouse, rat, monkey, and D. melanogaster. Similar results were obtained in our studies.

β-tubulin (cat# 2144S, Cell Signaling Technology). 539 citations for validation on the manufacture's website. Validated for western blot in CAD and C6 cells. Molecular weight is 52 kDa and reactive with human, mouse, rat, monkey, D. melanogaster, and bovine. Similar results were obtained in our studies.

Anti-Cyclin A (cat# sc-271682, Santa Cruz Biotechnology). 77 citations for validation on the manufacture's website. Validated for western blot in SK-BR-3, A-431, HeLa, K562, and HuT 78 cells. Molecular weight is 54 kDa and reactive with human. Similar results were obtained in our studies.

Anti-E2F1 (cat# sc-56661, Santa Cruz Biotechnology). 8 citations for validation on the manufacture's website. Validated for western blot in Jurkat, WEHI-23, SUP-T1, CCRF-CEM, and TK-1 cells. Molecular weight is 47 kDa and reactive with human. Similar results were obtained in our studies.

Anti-GAL9 (cat#348908, BioLegend). Validated for flow cytometry in human T-lymphoblastic leukemia cell line (MOLT4). Reactive with human, African Green Monkey, Baboon, and Cynomolgus. Similar results were obtained in our studies.

Anti-TIM3 (cat#130-126-004, Miltenyi Biotec). Validated for flow cytometry in human peripheral blood mononuclear cells (PBMCs) either left unstimulated or stimulated with CD3/CD28/IL-2 for 3 days. Reactive with human. Similar results were obtained in our studies.

Anti-CD20 Ab-1 (cat# MS-340-S0, Thermo Fisher Scientific). Validated for IHC/IF in Human cells. Similar results were obtained in our studies.

Eukaryotic cell lines

Policy information about cell lines

Cell line source(s)	All cell lines (Nalm6, REH, SEM, RCH-AcV, 697, preosteoblast, fibroblast, and OP9) used in this study were purchased from ATCC.
Authentication	Each cell lines was supplied with an authentication assurance at the time of purchase (using STR profiling).
Mycoplasma contamination	All cell lines used in these studies were tested for mycoplasma every six months using the PCR Mycoplasma Test Kit I/C (PromoKine; Cat No. PK-CA91-1096, Lot No. 447P085). All cell lines used in these studies were mycoplasma negative.
Commonly misidentified lines (See ICLAC register)	No commonly misidentified cell lines were used in this study.

Animals and other organisms

Policy information about studies involving animals; ARRIVE guidelines recommended for reporting animal research

Laboratory animals	Two-four month old C57BL/6 mice (The Jackson Laboratory) and NOG mice (Taconic; [genotype] sp/sp;ko/ko [nomenclature] NOD.Cg-Prkdcscid Il2rgtm1Sug/JicTac and [genotype] sp/sp;ko/y [nomenclature] NOD.Cg-Prkdcscid Il2rgtm1Sug/JicTac) were used in these studies. Both male and female mice were used for the experiments described in this study. Mice were housed at 68-72°F; relative humidity at 30-70%; 12/12 dark/light cycle; food (Bio-Serv; cat. no. F4301 for control diet and cat. no. S3282 for high-fat diet) and water ad libitum.
Wild animals	No experiments in this study.
Field-collected samples	No experiments in this study.
Ethics oversight	Animal studies presented in this manuscript were approved by EMORY UNIVERSITY: Institutional Animal Care and Use Committee under the approved protocol number DAR-3000013.

Note that full information on the approval of the study protocol must also be provided in the manuscript.

Human research participants

Policy information about studies involving human research participants

Population characteristics	De-identified diagnosis-obtained cryopreserved primary B-ALL cells were obtained for 26 pediatric patients through the Aflac Cancer and Blood Disorders Center Leukemia and Lymphoma Biorepository. De-identified age- and sex-matched healthy cryopreserved PBMCs were obtained from the Children's Healthcare of Atlanta and Emory University's Children's Clinical and Translational Discovery Core. Tables 2 and 3 in the manuscript contain demographics from the patient or donor who have submitted samples analyzed in our study including the presence of disease, age, gender, and race.
Recruitment	Every patient, healthy donor, or legal guardian of the patient/donor signed an informed consent prior to the collection of samples and de-identified banking of samples in the Aflac Cancer and Blood Disorders Center Leukemia and Lymphoma or Emory University's Children's Clinical and Translational Discovery Core biorepositories (approved protocol numbers IRB00034535 and IRB00089506, respectively). For patients receiving treatment for leukemia or lymphoma (male, female, all ethnicities, all ages), de-identified serum and PBMCs were banked at diagnosis, during treatment, and post-treatment. Similarly, samples of the same tissue origin were banked for healthy donors of the same demographics.

Ethics oversight	The use of human samples for this study received an IRB exemption from the Emory University Institutional Review Board given that our usage of de-identified samples does not meet the definition of research with "human subjects" or "clinical investigation" as set forth in Emory policies and procedures and federal rules.

Note that full information on the approval of the study protocol must also be provided in the manuscript.

Flow Cytometry

Plots

Confirm that:

- [x] The axis labels state the marker and fluorochrome used (e.g. CD4-FITC).
- [x] The axis scales are clearly visible. Include numbers along axes only for bottom left plot of group (a 'group' is an analysis of identical markers).
- [x] All plots are contour plots with outliers or pseudocolor plots.
- [x] A numerical value for number of cells or percentage (with statistics) is provided.

Methodology

Sample preparation	Human B-cell Cell Lines : Nalm6 cells were grown in RPMI1640 media (cat# 10-040-CV, Corning) supplemented with 10% fetal bovine serum (FBS, cat# S11550, Atlanta Biologicals). REH, SEM, RCH-AcV, and 697 cells were grown in RPMI1640 media supplemented with 20% FBS.<br><br>Murine Cell Lines: GFP-expressing OP-9 bone marrow stromal cells were maintained in Alpha-Minimum Essential Medium (αMEM, cat# 15-012-CV, Corning) supplemented with 20% FBS. Murine B-ALL cells (mB-ALL; BAML-Mig; GFP-expressing Bcr-Abl+ Arf-null) were grown in RPMI1640 media supplemented with 20% FBS.<br><br>In each case, murine or human B-ALL cell lines were cultured for 1-3 days in unconditioned media, stromal cell-conditioned media, or adipocyte-conditioned media prior to flow cytometric analysis.
Instrument	BD LSR II flow cytometer (BD Biosciences)
Software	Tree Star FlowJo software v10.6.1 (Ashland, Oregon)
Cell population abundance	In flow cytometry-based experiments, > 30,000 cells were collected per population. The purity of each population was determined by using a minimum of 5 colors, which allows precise population identification by direct or back-gating approaches.
Gating strategy	Only live cells were analyzed in experiments presented in this manuscript. Live cells were gated using forward scatter/ side scatter gating with a secondary validation using live cell dyes. Cells positive for surface antigens (e.g. Galectin-9) or stains (e.g. Annexin V/PI) were determine based on a combination of no stain, isotype, and unstimulated control samples.

- [x] Tick this box to confirm that a figure exemplifying the gating strategy is provided in the Supplementary Information.
